# Incidence of second malignancies in patients with thymic carcinoma and thymic neuroendocrine tumor

**DOI:** 10.1007/s00432-023-05522-3

**Published:** 2024-01-16

**Authors:** Guanghao Qiu, Fuqiang Wang, Yun Wang

**Affiliations:** 1 Center of Gerontology and Geriatrics, Laboratory of Metabolism and Aging Research, State Key Laboratory of Respiratory Health and Multimorbidity and National Clinical Research Center for Geriatrics, West China Hospital, Sichuan University, Chengdu, Sichuan China; 2 Department of Thoracic Surgery, West China Hospital, Sichuan University, No. 37, Guoxue Alley, Chengdu, 610041 Sichuan China

**Keywords:** Thymic carcinoma, Thymic neuroendocrine tumor, Standardized incidence ratio, Second malignancies

## Abstract

**Objectives:**

Thymic carcinoma and thymic neuroendocrine tumor (NET) are rare and are more likely to develop second malignancies. The purpose of this study was to explore the incidence and lifetime risk of second malignancies in thymic carcinoma and thymic NET.

**Methods:**

The standardized incidence ratio (SIR) and the age-adjusted cancer incidence of the thymic carcinoma and thymic NET patients with second malignancies were retrospectively calculated by using the Surveillance, Epidemiology, and End Results (SEER) database. Prognosis results were also determined by Kaplan–Meier analysis and Cox regression.

**Results:**

1130 patients with thymic carcinoma (73 patients had second malignancies) and 263 patients with thymic NET (19 patients had second malignancies) from 2000 to 2018 are included. Patients with thymic carcinoma (SIR: 1.36, 95% CI 1.08–1.69) and with thymic NET (SIR: 1.73, 95% CI 1.13–2.54) demonstrate an increased overall risk of developing second malignancies in various organ systems. The age-adjusted cancer incidence of second malignancies in patients with thymic carcinoma is 3058.48 per 100,000 persons (4178.46 per 100,000 persons in patients with thymic NET). Age at diagnosis is a significant risk factor for the development of second malignancies.

**Conclusion:**

The incidence of second malignancies in patients with thymic carcinoma and thymic NET is significantly higher than the patients in the normal population. The occurrence of second malignancies is not related to the use of different treatments. It is important to extend the follow-up period and add other screening methods.

## Introduction

The incidences of thymic malignancies that are rare tumors are less than 1% among adult cancers (Engels 2010). Thymoma, thymic carcinoma, and thymic neuroendocrine tumor (NET) are classified as thymic epithelial tumors (Venuta et al. 2010). Thymic carcinoma is a very rare malignant tumor, and its prognosis is worse than thymoma (Ahmad et al. 2015). Thymic NET is rarer and accounts for about 2–5% of thymic cancers, and its prognosis is poor (Gaur et al. 2010).

Previous studies have revealed the association between the incidence of second malignant tumors and thymoma (LeGolvan and Abell 1977; Gray and Gutowski 1979; Lewis et al. 1987; Couture and Mountain 1990; Wilkins et al. 1999; Welsh et al. 2000); because thymic carcinoma and thymic NET are rare, there are few studies on second malignancies arising after diagnosis. Badve suggested that 14 of 85 patients had a second malignancy including embryonal carcinoma, prostatic adenocarcinoma, small cell lung carcinoma, bladder carcinoma, testicular cancer, and so on (Badve et al. 2021). Hamaji found that the thymic carcinoma patients have a lower cumulative incidence of a second malignancy than thymoma patients. (Hamaji et al. 2019a). Apart from the limitation of small sample size, the study by Hamaji et al. also has the limitations of retrospective design, lacking some important data from the database and relatively short follow-up periods with fewer follow-up details (Hamaji et al. 2018, 2019b).

To overcome the problem of a small sample size and assess the incidence and lifetime risk, Surveillance, Epidemiology, and End Results (SEER) database was used to study second malignancies after being diagnosed with thymic carcinoma and thymic NET.

## Materials and methods

### Patients

The SEER 18-Registries SEER Research Plus Data (2000–2018, Nov 2020 Sub) could be analyzed. In this study, patients with thymic carcinoma were defined with ICD-O-3 codes 8010, 8020–8023, 8032–8033, 8052, 8070–8072, 8074, 8082, 8123, 8140, 8200, 8260, 8310, 8430, 8480, 8560, 8586, and 8589. Patients with thymic NET were defined with ICD-O-3 codes 8012, 8013, 8041, 8044, 8045, 8240, 8246, 8249, and 8574. The patients diagnosed with thymic carcinoma or thymic NET which was not the first cancer in the patient’s life were excluded. Standardized incidence ratio (SIR) which is the ratio of the number of observed cases divided by the number of expected cases was calculated to estimate the relative risk of second malignancy progression. The age-adjusted cancer incidence (per 100,000 persons) was calculated according to the 2000 US standard population census. The age-adjusted cancer incidence in the SEER Population was adopted using the average value of 2000 to 2018. Other parameters such as gender, age, race, and treatment were included. Overall survival (OS) was investigated by the Kaplan–Meier analysis.

The thymic carcinoma and thymic NET are rare, so given the limited sample size of second malignancies after diagnosed with thymic carcinoma and thymic NET, system groups such as respiratory system, endocrine system, and so on were employed to calculate the SIR and the age-adjusted cancer incidence. Furthermore, patients were grouped by different treatments.

### Statistical analysis

The two-sample *t* test was used to examine continuous variables. Statistical significance was considered as two-tailed *P* values less than 0.05. Pearson’s Chi-squared test statistic was used for analysis of categorical data. The log-rank test was performed to ascertain the differences between the Kaplan–Meier survival curves. The multivariable Cox regression models (including variables of age, sex, ethnicity, and treatment) were used to assess the prognostic effects. The statistical analysis was performed using SPSS 25 (IBM Corp., USA) and GraphPad Prism 8.0 (GraphPad Software, USA). SEER*Stat software (version 8.3.9.2) was utilized to calculate the SIR and the age-adjusted cancer incidence in the SEER population and extract the data of the study cohort from the SEER database. All authors are responsible for statistical review and verification.

## Results

The baseline characteristics are listed in Table 1, which summarizes the demographic and characteristics of the 1130 patients with thymic carcinoma and 263 patients with thymic NET from 2000 to 2018, with 48 (65.8%) patients ≥ 60, 41 (56.2%) male, and 50 (58.5%) white patients in the population of thymic carcinoma with second malignancies. Second malignancies were diagnosed 54.26 ± 44.58 months (median 39 months, ranging from 19 to 85.5 months) from the time of the thymic carcinoma diagnosis. The mean age of the thymic carcinoma at diagnosis was 59.57 ± 13.72 years (median age, 61 years old, ranging from 52 to 69 years) and the mean age of the second malignancies at diagnosis was 61.14 ± 12.30 years (median age, 64 years old, ranging from 50 to 69 years). Second malignancies were diagnosed 61.47 ± 52.89 months (median 37 months; ranging from 18 to 116 months) from the time of the thymic NET diagnosis. The mean age of the thymic NET at diagnosis was 54.54 ± 14.98 years (median age, 57 years old, ranging from 43 to 65 years) and the mean age of the second malignancies at diagnosis was 56.32 ± 13.12 years (median age, 59 years old, ranging from 48 to 64 years). Only 3 (4.1%) patients received no treatment among this population. 263 patients with thymic NET are included with 9 (47.4%) patients ≥ 60, 14 (73.7%) male, and 15 (78.9%) white patients in the population of thymic NET with second malignancies. Only one patient did not receive any treatment in this population. Seventy-three patients with thymic carcinoma and 19 patients with thymic NET have 79 and 26 s malignancies, respectively. Five patients have three second malignancies and two patients have four second malignancies in the thymic carcinoma population. Six patients had three second malignancies and one patient had four second malignancies in the thymic NET population.

**Table 1:** Characteristics of patients with thymic carcinoma and thymic neuroendocrine tumor

Characteristics	Second malignancies (thymic carcinoma, n = 1130)		p value	Second malignancies (thymic neuroendocrine tumor, n = 263)		p value
	Yes	No		Yes	No	
Age			0.068			0.586
< 60	25 (34.2%)	478 (45.2%)		10 (52.6%)	144 (59.0%)	
≥ 60	48 (65.8%)	579 (54.8%)		9 (47.4%)	100 (41.0%)	
Sex			0.300			0.538
Male	41 (56.2%)	658 (62.3%)		14 (73.7%)	163 (66.8%)	
Female	32 (43.8%)	399 (37.7%)		5 (26.3%)	81 (33.2%)	
Ethnicity			0.615			0.370
White	50 (58.5%)	698 (66.0%)		15 (78.9%)	171 (70.1%)	
Black	8 (11.0%)	160 (15.1%)		0	3 (29.4%)	
Other	15 (20.5%)	199 (18.8%)		4 (21.1%)	50 (20.5%)	
Treatment			0.006			0.337
No	3 (4.1%)	84 (7.9%)		1 (5.3%)	22 (9.0%)	
S or/and R	34 (46.6%)	331 (31.3%)		10 (52.6%)	116 (47.5%)	
C	5 (6.8%)	210 (19.9%)		0	30 (12.3%)	
S or/and R + C	31 (42.5%)	432 (40.9%)		8 (42.1%)	76 (31.1%)	

*S* surgery, *R* radiotherapy, *C* chemotherapy

The overall risk of developing second malignancies in patients with thymic carcinoma increased by 36% (SIR: 1.36, 95% CI 1.08–1.69) and with thymic NET increases by 73% (SIR: 1.73, 95% CI 1.13–2.54). Patients with thymic carcinoma have a particularly high risk of cancers of the respiratory and endocrine systems. Patients with thymic NET demonstrate a particularly high risk of cancers of the breast and endocrine system (Table 2).

**Table 2:** Standardized incidence ratio (SIR) of second malignancies in patients with thymic carcinoma and thymic neuroendocrine tumor

Cancer type	Thymic carcinoma		Thymic neuroendocrine tumor	
	SIR	95% CI	SIR	95% CI
All sites	1.36^#^ (n = 79)	1.08–1.69	1.73^#^ (n = 26)	1.13–2.54
Respiratory system	2.01^#^ (n = 18)	1.19–3.18	2.23 (n = 5)	0.73–5.21
Endocrine system	5.53^#^ (n = 5)	1.8–12.91	8.82^#^ (n = 2)	1.07–31.85
Female genital system	1.81 (n = 4)	0.49–4.64	–	–
All lymphatic and hematopoietic diseases	1.76 (n = 9)	0.81–3.34	0.75 (n = 1)	0.02–4.2
Urinary system	1.51 (n = 8)	0.65–2.98	2.08 (n = 3)	0.43–6.07
Digestive system	1.24 (n = 14)	0.68–2.08	1.34 (n = 4)	0.37–3.44
Male genital system	0.82 (n = 9)	0.38–1.56	0.59 (n = 2)	0.07–2.13
Breast	0.35 (n = 2)	0.04–1.25	4.07^#^ (n = 4)	1.11–10.43
Others	^a^(n = 10)		^b^(n = 5)	

^#^p < 0.05

^a^Others including oral cavity and pharynx (n = 1), mesothelioma (n = 1), soft tissue including heart (n = 1), eye and orbit (n = 1), melanoma of the skin (n = 1), Kaposi sarcoma (n = 1), and miscellaneous (n = 4)

^b^Others including oral cavity and pharynx (n = 2), melanoma of the skin (n = 1), brain (n = 1), and miscellaneous (n = 1)

As shown in Table 3, the age-adjusted cancer incidence in patients diagnosed with all-site neoplasms after thymic carcinoma is 3058.48 per 100,000, which is 6.58 times that of the age-adjusted cancer incidence in the SEER population (464.5 per 100,000). The age-adjusted incidence of cancer in patients diagnosed with thymic NET is 4178.46 per 100,000. The most common second malignancies in patients with thymic carcinoma are that of cancers of the respiratory system, all lymphatic and hematopoietic diseases, the urinary system, digestive system, and male genital system.

**Table 3:** Age-adjusted cancer incidence in patients with thymic carcinoma, thymic neuroendocrine tumor and in the SEER population (per 100,000 persons)

Cancer type	Incidence in the SEER population	Incidence in SEER patients after diagnosis of thymic carcinoma	Incidence in SEER patients after diagnosis of thymic neuroendocrine tumor
All sites	464.5	3058.48	4178.46
Respiratory system	63.0	674.62	945.34
Endocrine system	12.9	97.96	331.07
Female genital system	26.5	282.85	-
All lymphatic and hematopoietic diseases	42.5	326.39	153.35
Urinary system	36.3	360.48	480.22
Digestive system	84.7	561.94	280.23
Male genital system	65.6	380.02	412.19
Breast	68.3	58.70	480.22
Others	64.7	315.52	1095.84

Age at diagnosis is a significant risk factor for the development of second malignancies after the diagnosis of thymic carcinoma in multivariable analyses (Table 4). The time of diagnosis of the second malignancies is set as the endpoint.

**Table 4:** Results of multivariable analysis using the COX proportional hazards model of the study population related to factors influencing the development of second malignancies after the diagnosis of thymic carcinoma and thymic neuroendocrine tumor

Variable	Second malignancies (thymic carcinoma)		Second malignancies (thymic neuroendocrine tumor)	
	HR (95% CI)	P value	HR (95% CI)	P value
Age				
< 60	–	–	–	–
≥ 60	2.114 (1.276–3.502)	0.004	1.848 (0.696–4.910)	0.218
Sex				
Male	–	–	–	–
Female	1.052(0.657–1.684)	0.832	1.004(0.355–2.838)	0.994
Ethnicity				
White	–	–	–	–
Black	1.007 (0.470–2.156)	0.986	^a^	^a^
Other	1.193 (0.665–2.141)	0.554	0.989 (0.307–3.189)	0.985
Treatment				
No	–	–	–	–
S or/and R	1.058 (0.323–3.464)	0.925	0.573 (0.070–4.690)	0.603
C	0.610 (0.145–2.567)	0.500	^a^	0.979
S or/and R + C	1.101 (0.335–3.622)	0.875	1.310 (0.148–11.592)	0.808

*S* surgery, *R* radiotherapy, *C* chemotherapy

^a^Invalid value

**Fig. 1:**
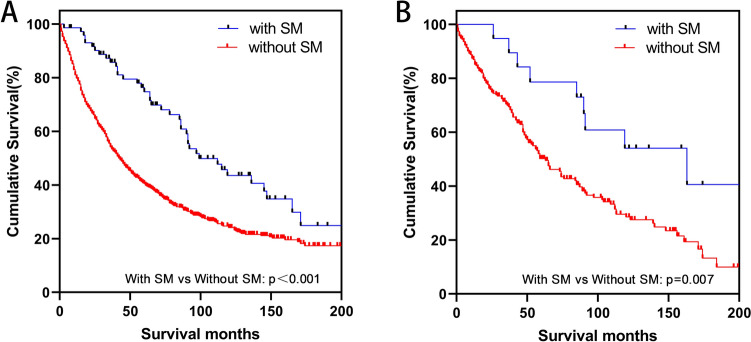
Kaplan–Meier curve of the comparison of overall survival in patients with and without second malignancies (**A** patients with thymic carcinoma, **B** patients with thymic neuroendocrine tumor) in the total study population (*SM* second malignancies)

In Fig. 1, the thymic carcinoma patients with second malignancies show a statistically significant difference to the patients without second malignancies in the prognosis (OS: *p* < 0.001). The prognosis of the thymic NET patients has similar results (OS: *p* = 0.007). The median survival time is 44 months (95% CI 38.95–49.05) in the total thymic carcinoma population. The thymic carcinoma patients with second malignancies show a median survival time of 99 months (95% CI 68.88–129.12). The thymic carcinoma patients without second malignancies have a median survival time of 41 months (95% CI 36.58–45.42). Similarly, the median survival times for the total thymic NET population, the thymic NET with second malignancies population, and the thymic NET without second malignancies population are 65 months (95% CI 51.04–78.96), 163 months (95% CI 59.68–266.32), and 59 months (95% CI 45.00–73.00), respectively.

**Fig. 2:**
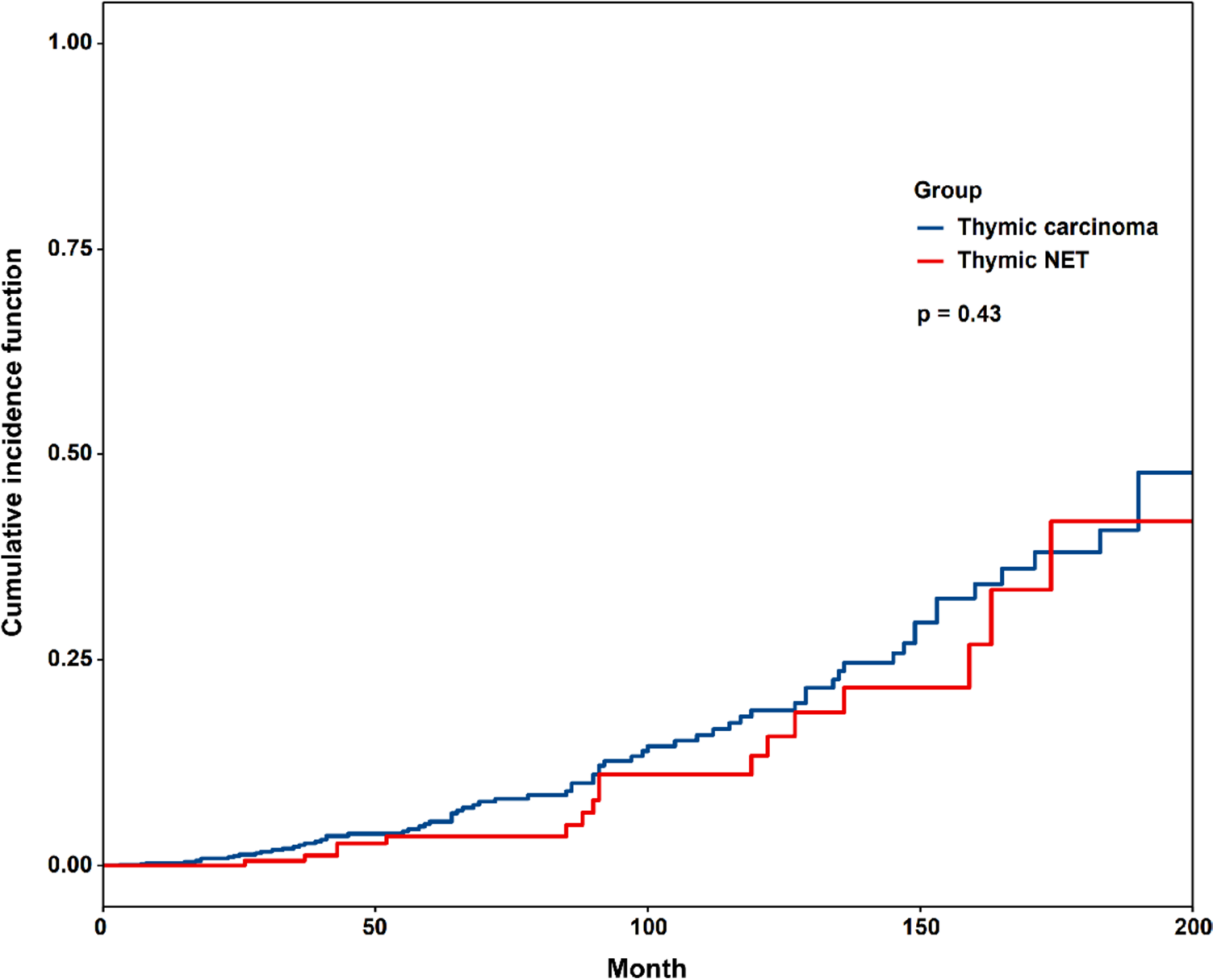
Cumulative incidence function based on thymic carcinoma and thymic neuroendocrine tumor

Cumulative incidence function (CIF) values based on thymic carcinoma and thymic NET are shown in Fig. 2. In patients with thymic carcinoma, CIF of second malignancies is 2.39% at 5 years and 4.69% at 10 years. In patients with thymic NET, CIF of second malignancies is 1.52% at 5 years and 4.18% at 10 years.

## Discussion

Thymic carcinoma is a relatively rare malignant tumor that accounts for less than 0.01% of all malignancies diagnosed each year. The rarity of the disease has become an important limiting factor in determining its natural history. Thymic NET that is rarer than thymic carcinoma has the same problem. We attempted to circumvent this limitation by using the SEER database which gathers and publishes data on cancer incidence and lifetime risk for about 28% of the American population.

SIR values for second malignancies in all sites and for each system of cancers in the SEER patients were calculated after diagnosis of thymic carcinoma and thymic NET. This finding indicated that thymic carcinoma has a significantly increased risk for the development of the respiratory system, endocrine system, and all sites. Furthermore, thymic NET shows a distinct increased risk for the development of breast, endocrine system, and all sites. The risk of second malignancies is not increased by different therapies for thymic carcinoma and thymic NET. The present study is the largest of its kind examining the link between thymic carcinoma or thymic NET and second malignancies, looking at cancer incidence after diagnosis.

According to the RARECARE project in Europe, Siesling et al. reported that thymic epithelial tumors have an age-standardized incidence of 0.17 per 100,000 people at risk (Siesling et al. 2012). Based on the data from The Netherlands Cancer Registry Database and The Netherlands National Pathological Archives (PALGA), de Jong et al. found that the incidence of thymic carcinoma is 0.03–0.06 per 100,000 population at risk in The Netherlands (de Jong et al. 2008). According to Gaur et al., the incidence of thymic NET is 0.02 per 100,000 people at risk (Gaur et al. 2010). Yi-Ting Yen et al. found the overall incidence of second malignancies in patients with thymic carcinoma as being 12.2% (10 of 82) (Yen et al. 2011).

We believe that only patients who live longer or with early stage are more likely to have second malignancies, which results in a longer survival period for the patients with second malignancies in Fig. 1.

Existing theories are unable to explain fully those second malignancies diagnosed before thymic carcinoma and thymic NET. There are some possibilities to explain that phenomenon in our opinion. First, the second malignancies diagnosed before thymic malignancies are primary tumors, which are not affected by the thymus. Second, the thymic environment may have already changed a long time before the diagnosis of thymic malignancies, which may be the cause of second malignancies before the thymic malignancies. The reason why thoracic malignancy (as the second malignancy) is common in thymic carcinoma may be the frequent survey of intrathoracic recurrence by chest CT. Our results reveal that thymic NET has higher incidence of endocrine malignancy, which may be due to multiple neuroendocrine neoplasia syndrome associated with thymic carcinoid cases (Ferolla et al. 2005). Thymic NET is may associated with multiple endocrine neoplasia type 1. Meanwhile, a study by NEJM shows that patients who have undergone thymectomy are associated with an increase in autoimmune diseases (Kooshesh et al. 2023). What’s more, age is associated with the hazard of time to onset of second malignancies, so the increase of malignancy incidence with aging may be an important reason.

According to the National Comprehensive Cancer Network guidelines, thymic carcinoma patients have a follow-up period of 5 years and surveillance chest CT should be undertaken to identify disease recurrence after TETs resection every 6 months (Oncology NCPGi. 2017). Patients’ second malignancies were found in different places and at different times in our investigation, as in earlier studies of thymoma (Pan et al. 2001; Evoli et al. 2004; Owe et al. 2010; Weksler et al. 2012; Filosso et al. 2013; Kamata et al. 2017). Because the location of the extrathymic neoplasm is uncertain, adding abdominal CT or other screening methods are options that can be considered.

This study has limitations: although a large database has been applied, the sample sizes remain small given the rareness of thymic carcinoma and thymic NET, thus when calculating the incidence, we used the approach of age adjustment to generalize age-specific rates. However, there was still a lack of patients in some age groups. We introduced the static population data of the United States in 2000 as the standard population, which cannot describe population changes more accurately. Some parts of the data such as stage, grade, and whether cases are accompanied by the paraneoplastic syndrome are missing, which affect the results. Due to the limitations related to the size of the database, the benign tumors cannot be included, which may render our results inaccurate.

## Conclusion

Second malignancies are more common in patients with thymic carcinoma and thymic NET, especially in the respiratory system and endocrine system (patients with thymic carcinoma) and in the endocrine system and breast (patients with thymic NET). The age-adjusted cancer incidence of second malignancies increased after being diagnosed with thymic carcinoma and thymic NET. The increased incidence was unrelated to type of treatment delivered. The high incidence of second malignancies makes it important to extend the follow-up period and add abdominal and chest CT periodically. To confirm the association of second malignancies and thymic carcinoma or thymic NET, a larger sample size in such studies is warranted in the future.

## Data Availability

The datasets generated during and/or analysed during the current study are available from the corresponding author on reasonable request.

## Abbreviations

NET: Thymic neuroendocrine tumor
SIR: Standardized incidence ratio
SEER: Surveillance, Epidemiology, and End Results
OS: Overall survival
MG: Myasthenia gravis
S: Surgery
R: Radiotherapy
C: Chemotherapy
